# The Resistance to Lethal Challenge with *Ostreid herpesvirus-1* of Pacific Oysters (*Crassostrea gigas*) Previously Exposed to This Virus

**DOI:** 10.3390/v15081706

**Published:** 2023-08-08

**Authors:** Olivia M. Liu, Paul M. Hick, Richard J. Whittington

**Affiliations:** 1 Sydney School of Veterinary Science, Faculty of Science, The University of Sydney, 425 Werombi Road, Camden, NSW 2570, Australia; olivia.liu@aff.gov.au (O.M.L.); paul.hick@dpi.nsw.gov.au (P.M.H.); 2 Biosecurity Animal Division, Australian Government Department of Agriculture, Fisheries and Forestry, Canberra, ACT 2601, Australia; 3 Elizabeth Macarthur Agricultural Institute, Woodbridge Road, Menangle, NSW 2568, Australia

**Keywords:** *Ostreid herpesvirus*, immunity, resistance, susceptibility, carrier state, reactivation, transmission, experimental infection, oyster, disease control

## Abstract

Pacific oyster (*Crassostrea gigas*) aquaculture has been economically impacted in many countries by Pacific oyster mortality syndrome (POMS), a disease initiated by *Ostreid herpesvirus 1*. The objectives of this study were to determine whether naturally exposed, adult *C. gigas* could act as reservoirs for OsHV-1 and explain the recurrent seasonal outbreaks of POMS and to test whether or not they were resistant to OsHV-1. In a laboratory infection experiment using thermal shock, OsHV-1 replication was not reactivated within the tissues of such oysters and the virus was not transmitted to naïve cohabitating spat. The adult oysters were resistant to intramuscular injection with a lethal dose of OsHV-1 and had 118 times lower risk of mortality than naïve oysters. Considered together with the results of other studies in *C. gigas*, natural exposure or laboratory exposure to OsHV-1 may result in immunity during subsequent exposure events, either in the natural environment or the laboratory. While adult *C. gigas* can carry OsHV-1 infection for lengthy periods, reactivation of viral replication leading to mortality and transmission of the virus to naïve oysters may require specific conditions that were not present in the current experiment. Further investigation is required to evaluate the mechanisms responsible for resistance to disease in oysters previously exposed to OsHV-1, whether immunity can be exploited commercially to prevent POMS outbreaks and to determine the source of the virus for recurrent seasonal outbreaks.

## 1. Introduction

The commercial production of Pacific oysters (*Crassostrea gigas*) in many countries has been severely impacted by Pacific oyster mortality syndrome (POMS), a multifactorial disease caused by *Ostreid herpesvirus-1* [1,2,3]. Belonging to the family *Malacoherpesviridae* within the order *Herpesvirales* [4,5], OsHV-1 has diverse genotypes comprising both pathogenic and apparently non-pathogenic varieties. OsHV-1 µVar, first detected in France in association with mass mortalities in cultured *C. gigas* [6], has been reported commonly in Europe since about 2008 and is considered to be a significant pathogen. The World Health Organisation for Animal Health (OIE) defined the µVar genotype and several closely related variants as “OsHV-1 microvariants” [7]. While OsHV-1 µVar was thought to be responsible for outbreaks of POMS in Australia, further research revealed a range of genotypes, none of which were microvariants [8,9].

Affecting all age and size classes of *C. gigas*, OsHV-1 causes mortalities of between 40% and 70% in adult oysters and 60% and 100% in oysters <1 year of age [10,11,12,13,14,15]. The severity of the disease is influenced by a range of host and environmental factors [16,17,18]. Experimentally infected oysters, injected intramuscularly with a semi-purified homogenate containing OsHV-1, exhibited an exponential increase in OsHV-1 DNA 48 h post injection, died within 48 to 96 h with viral concentrations of 1 × 10^5^ to 3 × 10^7^ OsHV-1 DNA copies mg^−1^ tissue and there was a clear dose–response effect [19,20]. However, a small percentage of injected oysters survived and OsHV-1 DNA was detected in the gill and mantle at 1.1 × 10^3^ DNA copies mg^−1^ tissue 10 days post injection [20]. A reduction in viral gene transcription and viral DNA load has been reported at 5 days post injection, indicating the end of active infection and control of viral replication within the host [21], with timing influenced by water temperature [22]. Oysters that survived either natural OsHV-1 exposure or experimental infection have been reported in many studies [19,20,23,24,25]. OsHV-1 has been detected in the tissues of apparently healthy *C. gigas* ranging in age from spat <1 year old to adults 7 years of age in France, Korea, Japan and Australia [24,26,27,28]. Detailed observations of this phenomenon were made in a longitudinal study in Australia in which the hemolymph of 150 individually identified *C. gigas* from three separate cohorts that had survived mass mortality events was repeatedly sampled over 15 months [24]. The prevalence of OsHV-1 in the youngest cohort that had been exposed for the first time a few months earlier declined from 30% to <12%, while the prevalence in two older cohorts that had been exposed more than once during previous seasons was consistently less than 10% [24]. OsHV-1 DNA was detected at low levels (<10^4^ DNA copies per mg tissue or µL hemolymph) in these healthy oysters [24]. This was evidence that OsHV-1 was not being maintained at or amplified to the high levels that are consistent with clinical disease [20,29]. These features of OsHV-1 are similar to other aquatic herpesviruses such as koi herpesvirus, channel catfish virus and cyprinid herpesvirus 3 [30,31,32,33] and lead to questions about immunity, latent infection and reactivation, which are features of herpesvirus infections in higher animals [34]. Water temperature is important for OsHV-1 disease expression [35,36,37,38] and temperature shock has been used to reactivate OsHV-1 replication in infected *C. gigas* in France. High levels of mortality (30% to 70%) were reported in thermally shocked oysters, along with increased viral quantities and transmission of the virus to naïve, cohabitating *C. gigas* [35,39,40].

Due to persistent OsHV-1 infection, apparently healthy *C. gigas* could be classified as carriers, raising the possibility that they could be the reservoir of infection for the recurrent outbreaks of POMS that are commonly observed in the warmer months each year. However, the low viral quantities detected in their tissues and the fact that the levels of virus do not increase seasonally but actually decline over time suggests that viral replication is either controlled or cleared [21,24] and that other reservoirs exist for the virus [41]. The resistance to OsHV-1 that exists within populations of oysters that have gone through disease outbreaks has provided an opportunity for genetic selection and breeding for resistance [11,42,43], but other opportunities may exist to exploit immunity to prevent disease at commercial scale.

The aims of this study were (1) to assess whether OsHV-1 replication can be reactivated within the tissues of previously exposed adult (PEad) oysters by increasing water temperature, (2) to assess whether thermally shocked PEad oysters can transmit OsHV-1 to naïve cohabitating spat, and (3) to assess whether PEad oysters are susceptible to reinfection with OsHV-1, or clinical disease, using an intramuscular injection model.

## 2. Materials and Methods

### 2.1. Oysters

All batches/groups of oysters were certified as negative for OsHV-1 using quantitative PCR (qPCR) by the competent government authority prior to their transfer from the hatchery of origin in Tasmania to New South Wales (NSW) and again after arrival in NSW by the University of Sydney using the qPCR protocol described below prior to use in each experiment.

#### 2.1.1. Previously Exposed Adult Oysters (PEad)

One hundred and forty-four oysters that had been previously naturally exposed to OsHV-1 under field conditions were recruited from a population of experimental oysters maintained in the Georges River estuary, NSW [15,24]. Briefly, the oysters originated from two batches of single-seed hatchery-reared *C. gigas* spat (batches SPL14B and SPL13B, Shellfish Culture, Hobart, Tasmania). The oysters were maintained in the OsHV-1-free Shoalhaven River estuary, NSW, before being transferred to the Georges River estuary in January 2015 when the two batches were 8 and 18 months old, respectively (see Table 1 in Evans, Hick and Whittington [24]).

This population of oysters was exposed to OsHV-1 and consisted of the survivors of mass mortality outbreaks in the Georges River in January–April 2015, November–December 2015 and January–June 2016 [24]. During the first exposure, cumulative mortality was 26.8% to 98.8% (range) [15], viral concentrations in the gill/mantle of dead oysters were 3.96 × 10^3^ ± 7.35 × 10^1^ to 2.87 × 10^5^ ± 2.94 × 10^1^ DNA copies per mg tissue [15,24] and the prevalence of OsHV-1 in surviving oysters was 14.5% (95% CI: 8.7–22.2%) [15]. Fifty surviving oysters from this population were sampled next in November 2015 and again in August 2016; OsHV-1 DNA was detected at a prevalence of 2% and with viral loads below the level of quantification [24]. These oysters are referred to hereafter as “previously exposed adult” (PEad) oysters. They were recruited to this study in September 2016 at the age of approximately 27–37 months and 123.6 ± 18.37 mm shell length, but by then, the two hatchery batches were not distinguishable.

#### 2.1.2. Non-Exposed Adult Oysters (NEad)

Single-seed hatchery-reared *C. gigas* spat (batch SPL13A; Shellfish Culture, Hobart, Australia) were maintained at a site in Patonga Creek NSW, known to be free of OsHV-1 mortality events [44] for 2.5 years before being transferred to the University of Sydney laboratory at the age of 36 months and 75.0 ± 8.6 mm shell length. They are referred to hereafter as non-exposed adult (NEad) oysters.

#### 2.1.3. Non-Exposed Spat (NEsp)

One batch of hatchery-reared single-seed *C. gigas* spat (batch SPL15AT; Shellfish Culture, Hobart, Australia) was split and reared as two groups prior to use in this study. Group 1, referred to hereafter as “non-exposed spat group 1” (NEsp1), were transferred from the hatchery to the University of Sydney laboratory and maintained on a diet of concentrated marine microalgae (Instant Algae^©^ Shellfish diet 1800, Reed Mariculture, Campbell, CA, USA) in a 300 L aquarium connected to biofiltration and held at 22 °C ± 1 °C for 12 months prior to use in experiment 1. NEsp1 were approximately 2 mm ± 1 mm in shell length on arrival at the laboratory and 4 mm ± 1 mm in shell length upon recruitment to experiment 1 at approximately 15 months of age. Group 2, referred to hereafter as “non-exposed spat group 2” (NEsp2), were transferred from the hatchery to Patonga Creek, NSW. They were maintained at the Patonga Creek site for 12 months before being transferred to the University of Sydney laboratory at approximately 15 months of age and 67.5 ± 8.9 mm in shell length.

### 2.2. Aquarium Facility

**Figure 1:**
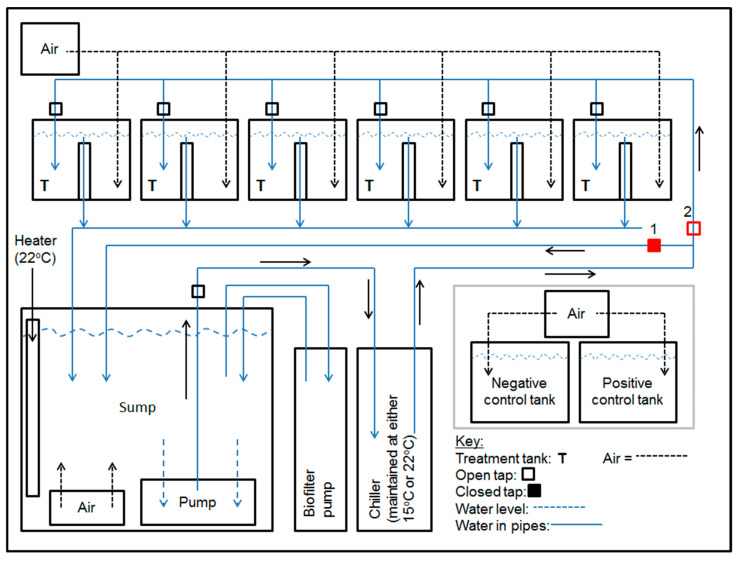
One of four identical recirculation systems in which oysters were housed. Each consisted of six 20 L tanks, a 250 L sump, a canister biofilter, a water chiller and an external air pump. During feeding, the positions of taps 1 and 2 were reversed to cause water to bypass the treatment tanks; tank water was aerated during feeding. An aquarium heater was added to the sump of systems 1, 2 and 3 on days 7 to 12 in experiment 1. Two aerated 20 L control tanks were included in experiment 2. Schematic not to scale.

The experiments were undertaken in a physical containment level 2 aquatic animal facility at the University of Sydney, Camden NSW. Four separate recirculation systems were used. Each system had a 250 L sump connected to a cannister biofilter (Fluval406), an aquarium chiller (15 °C; HC-300A Hailea) and an aquarium heater (Figure 1). The total volume of each system was approximately 400 L. Each system was supplied with artificial seawater (Red Sea salt, 30 ± 1.0 ppt; prepared with unfiltered, dechlorinated water from the municipal supply) maintained at pH 8.0–8.2 and ammonium, nitrite and nitrate levels of <0.25 ppm. The water temperature was adjusted according to the experiment (see below). Water temperature was recorded every 15 min using data loggers (1921 G, Thermocron^®^, Castle Hill, Australia), each enclosed in a thin-walled, waterproof, polypropylene tube, placed at the bottom of the sump or control tank. A photoperiod of 12 h light and 12 h dark was used. Oysters were fed twice daily (10:00 and 14:00) with a maintenance diet of concentrated marine microalgae (2 mL per tank; Instant Algae^©^ Shellfish Diet 1800, Reed Mariculture, Campbell, CA, USA). During feeding, each tank was isolated from recirculation and biofiltration for 1 h, but was aerated (Figure 1).

### 2.3. Experiment 1

Experiment 1 assessed whether OsHV-1 replication can be reactivated in previously exposed adult (PEad) oysters after thermal shock and whether thermally shocked PEad oysters can transmit OsHV-1 to naive cohabitating spat.

**Figure 2:**
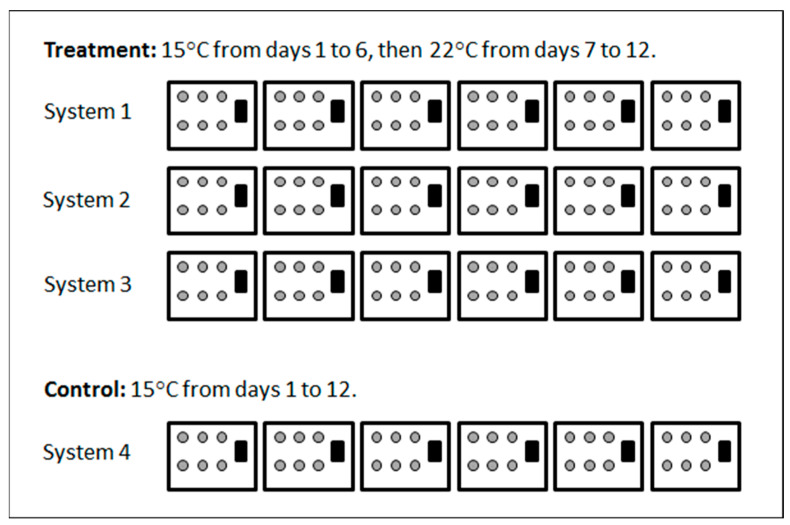
Schematic representation of the design for experiment 1, displaying the distribution of previously exposed adult oysters (PEad) (grey circles, 6 per tank) and the non-exposed spat (NEsp1) (100 per tank enclosed in one mesh sock) (black rectangles).

Previously exposed adult oysters (PEad) were depurated at 15 °C for 17 h in a 1000 L flow-through tank at the Georges River prior to transfer to the laboratory in insulated boxes. They were placed into the four systems and held at 15 °C. Six oysters were included per 20 L tank (Figure 2). To test for transmission of OsHV-1, one 100 × 100 mm polypropylene envelope with 2 mm^2^ mesh containing 100 non-exposed spat (NEsp1) was placed into each tank with the PEad (Figure 2). Prior to transfer, the water temperature in the spat holding tank was lowered from 22 °C to 15 °C over 48 h. Systems 1, 2 and 3 were maintained at a target temperature of 15 °C from days 1 to 6. On day 7, the temperature was raised to 22 °C over 4 h and maintained at this target temperature from days 7 to 12. System 4 was the control, maintained at 15 °C from days 1 to 12 (Figure 2).

All oysters were individually examined twice daily. On days 6, 9 and 12, water samples (15 mL) were collected from the sump of each system and from each tank; these samples were frozen at −20 °C until processing. On day 12, all NEsp1 spat in systems 1,2 and 3 and all of the PEad in system 4 were sampled. In addition, on day 12, two PEad were randomly selected and sampled from each tank in systems 1, 2 and 3; the remaining PEad were left in situ for use in experiment 2. Sampled oysters were stored at −80 °C until tissue dissection.

### 2.4. Experiment 2

Experiment 2 assessed whether previously exposed adult (PEad) oysters are susceptible to infection with OsHV-1, using an intramuscular injection model.

**Figure 3:**
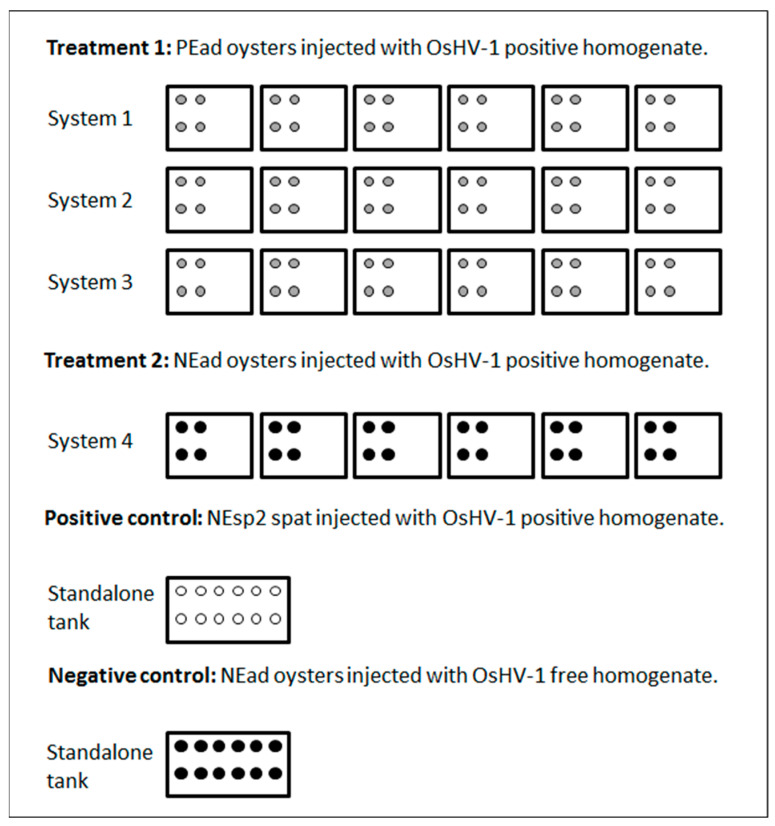
Schematic representation of the design for experiment 2, displaying the distribution of previously exposed adult oysters (PEad) (grey circles; 4 per tank), non-exposed adult oysters (NEad) (black circles; 4 per tank in system 4 and 12 in the negative control tank) and non-exposed spat (NEsp2) (white circles, 12 in the positive control tank).

After the first experiment, all of the PEad that remained in systems 1, 2 and 3 were used in experiment 2 in their original tanks (n = 72; 4 per tank), while non-exposed adult oysters (NEad) were added to system 4 (n = 24, 4 per tank) (Figure 3). Twelve non-exposed spat (NEsp2) were placed into a separate 20 L tank without recirculation or biofiltration as a positive control for the virulence of the OsHV-1 inoculum, while 12 NEad were placed into a similar tank as the negative control (Figure 3). The water temperature in each system and in the control tanks was maintained at 22 °C.

OsHV-1 inoculum and a negative control inoculum were made from cryopreserved stocks of 0.2 µm filtered tissue homogenates that had been prepared from gill and mantle tissues from OsHV-1 infected and non-infected *C. gigas*, respectively, as previously described [45]. The cryopreserved stocks were thawed on wet ice in a class 2 biosafety cabinet for 5 min and diluted 1:100 with 0.2 µm filtered artificial seawater. The OsHV-1 positive homogenate contained approximately 5.07 × 10^4^ OsHV-1 DNA copies per 100 µL. The negative control homogenate was confirmed free of OsHV-1 by real-time qPCR.

For inoculation, oysters were removed from water for 24 h, relaxed in MgCl_2_ (50 g L^−1^) at 24 °C for 4 h, rinsed briefly in artificial seawater (30 ppt) and injected in the adductor muscle using a sterile 25-gauge needle. Negative controls were injected with 100 µL of negative control inoculum. NEad and NEsp2 were injected with 100 µL of OsHV-1 inoculum, while PEad oysters in systems 1, 2 and 3 were injected with 500 µL of the OsHV-1 inoculum due to their larger size. Negative control oysters were injected before the other oysters and were kept separate from them at all times. After injection, oysters were kept out of the water for approximately 5 min prior to being placed back into their respective tanks.

Oysters were checked twice daily at 09:00 and 15:00 and the number of live and dead oysters was counted. Dead oysters were removed from the tanks and placed at −80 °C. On days 2, 5 and 8 after injection, water samples (15 mL) were collected from the sump of each system and from every tank; these were frozen at −20 °C until processing. On day 4 after injection, two live oysters from each tank in each system and six live oysters from each control tank were randomly sampled. On day 10, all remaining oysters were sampled. All these oysters were placed at −80 °C until tissue dissection.

### 2.5. Detection of OsHV-1 DNA

A piece of gill and mantle (0.08–0.12 g) was dissected from larger oysters using sterile disposable dissecting instruments and placed into a 1.5 mL tube containing 1 mL molecular grade water (Ultrapure^TM^, ThermoFisher, Waltham, USA) and 0.4 g of silica–zirconia beads (Daintree Scientific, St Helens, Australia). Spat were processed in pools as previously described [44]. Samples were frozen at −80 °C until homogenisation in a TissueLyser II machine (Qiagen) [25,44]. Seawater samples were processed as previously described [46]. All the resulting clarified tissue homogenate supernatants and processed seawater samples were stored at −80 °C. Nucleic acids were purified from these samples (50 µL for tissue samples; 200 µL for seawater samples) using an Ambion MagMax Viral isolation kit according as described [45]. Nucleic acid preparations were stored at −20 °C until tested in duplicate in 25 µL reaction volumes using probe-based real-time qPCR adapted from Martenot et al. [47] as described [46]. The quantification limit of the assay was 12 viral copies per PCR reaction. Samples that satisfied the criteria for detection [46] but with a Ct value below the quantification limit of the assay were described as positive below the limit of quantification (bloq).

### 2.6. Statistical Analysis

Cumulative mortality was calculated allowing for the number of live oysters sampled [14]. Survival analyses were conducted to compare the mortality of oysters between treatment groups using the Kaplan–Meier and Cox’s hazard models in GenStat (15th edition, ©2000–2015 VSN International Inc., Hemel Hempstead, United Kingdom) and SAS (©2002–2012 by SAS Institute Inc., Cary, NC, USA), respectively. Oysters sampled live were considered to have been censored at the respective observation times. All oysters surviving to the end of the experiment were also censored.

## 3. Results

### 3.1. Experiment 1

The seawater temperatures recorded in each system were close to the design targets (Table 1). No mortality was observed in any of the previously exposed adult oysters (PEad) or non-exposed spat (NEsp1) during the experiment. On day 12, five live PEad oysters tested positive for OsHV-1 DNA at low viral concentrations (bloq) (Table 1). All NEsp1 spat tested negative for OsHV-1 DNA, as did all the seawater samples.

### 3.2. Experiment 2

**Figure 4:**
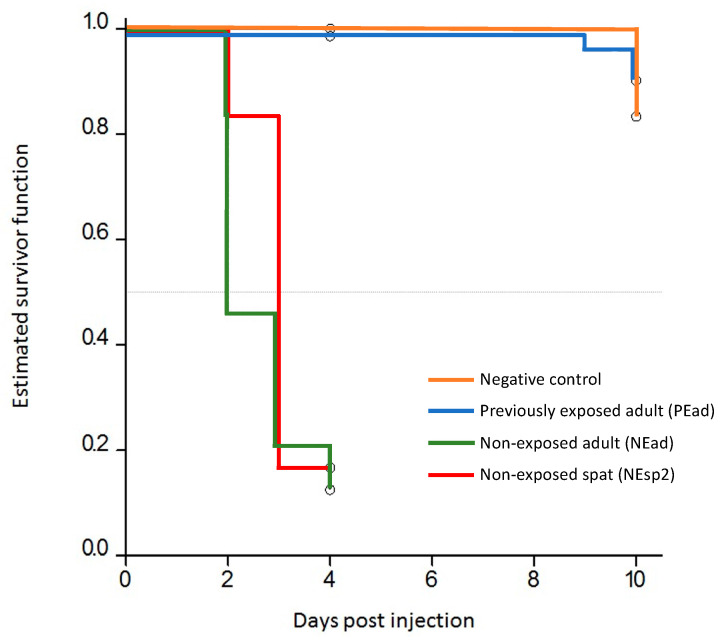
Kaplan–Meier survival curve for previously exposed adult oysters (PEad), non-exposed adult oysters (NEad) and non-exposed spat (NEsp2) injected with OsHV-1 in experiment 2. No mortality was observed in the negative control oysters. Live oysters were sampled on days 4 and 10 post injection (circles).

The seawater temperatures during the experiment were close to the design target of 22 °C (Table 2). Mortality began in non-exposed adult oysters (NEad) and the positive control spat (NEsp2) on day 2 post injection (pi) and continued until day 4 pi, reaching cumulative mortality of 91.7% and 83.3%, respectively (Figure 4). Mortality in previously exposed adult oysters (PEad) was much less. It began on day 2 pi when one dead oyster was observed in system 2. Two died in system 3 between days 7 and 9 pi, and a fourth died in system 1 on day 10 pi. The cumulative mortality in PEad oysters in systems 1, 2 and 3 was 8.3%, 16.7% and 4.2%, respectively; the total across the three systems was 9.8% (Figure 4). No mortality was observed in the negative control oysters during the experiment (Figure 4). Survival analysis indicated a significant difference between the treatments (*p* < 0.001). The median time to death for the NEad oysters was 2–3 days. The median time to death for the positive control spat was 3 days. Median times to death could not be estimated for the PEad oysters or the negative control oysters. NEad oysters had a hazard of death 118.5 times that of PEad oysters (95% CI: 23.8, 2175.1).

**Figure 5:**
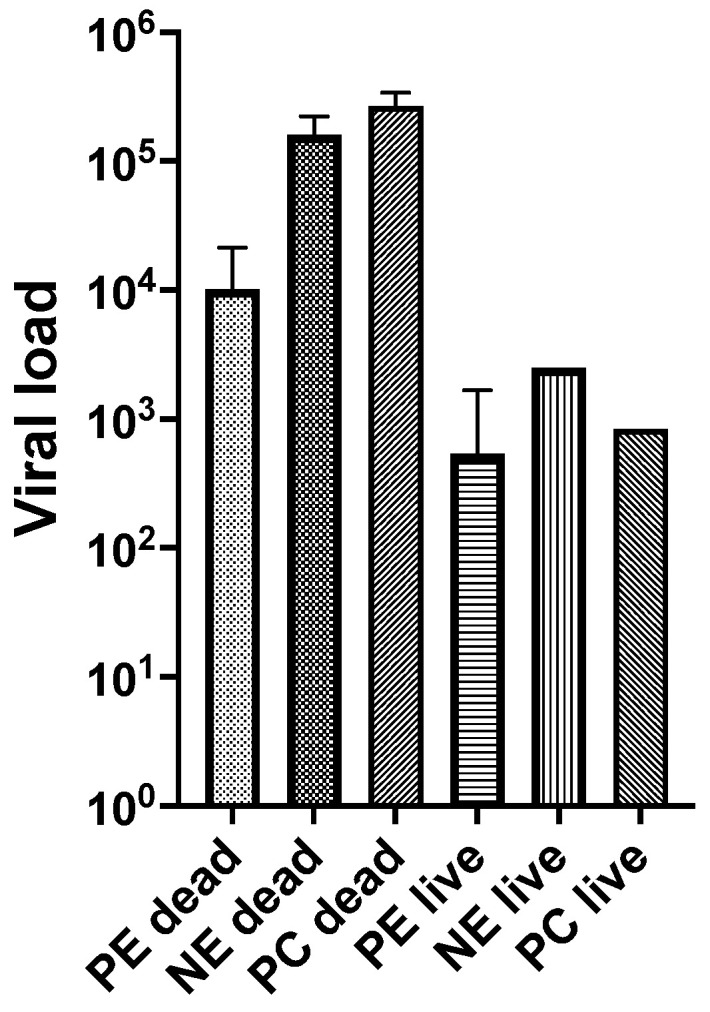
Viral load in previously exposed (PE), non-exposed (NE) and positive control (PC) oysters according to whether they were dead or live at the time of sampling. Bars represent mean and range OsHV-1 DNA copies/mg tissue from data in Table 3.

The OsHV-1 DNA concentrations in gill and mantle tissues are summarised in Table 3. The 21 oysters in the NEad group that died between days 2 and 4 pi had approximately 10^5^ DNA copies mg^−1^ tissue (geometric mean), while the four oysters in the PEad group that died between days 2 and 10 had 10^2^ to 10^4^ DNA copies mg^−1^ tissue. On day 4, OsHV-1 DNA was detected in gill/mantle tissues of 30/36 live PEad oysters and the viral load was approximately 10^2^ to 10^3^ DNA copies mg^−1^ tissue, but by day 10, when 26/32 live oysters contained detectable OsHV-1 DNA, the viral load had dropped to 10^1^ to 10^2^ DNA copies mg^−1^ tissue (Table 3); the proportions of infected oysters at days 4 and 10 were not significantly different (*p* > 0.05). The few remaining live oysters on day 4 pi in the NEad and positive control groups had approximately 10^3^ DNA copies mg^−1^ tissue. Viral loads for dead and live oysters in each group (ignoring day of sampling) are presented in Figure 5. While dead oysters had at least one log higher viral loads than oysters that were alive when sampled, those in the non-exposed group had higher viral loads than those in the previously exposed group. OsHV-1 DNA was not detected in any of the oysters in the negative control group.

On day 2 pi, low concentrations (bloq) of OsHV-1 DNA were detected in water from the positive control tank and in five of six tanks and the sump of system 4 (NEad oysters; Figure 3) in which most oysters subsequently died; no further water samples were collected. On day 8 pi, a low concentration (bloq) of OsHV-1 DNA was detected in the water from one tank of system 3 (PEad oysters; Figure 3); all water samples collected on days 2 and 5 pi were negative for OsHV-1 DNA. None of the water samples from systems 1 and 2 or the negative control tank had detectable OsHV-1 DNA.

## 4. Discussion

The first objective of this study was to determine whether OsHV-1 replication can be reactivated within the tissues of adult oysters that carry the virus, leading to mortality, and if so, whether OsHV-1 would be then transmitted to naïve cohabitating spat. If these features could be demonstrated, then it might help explain the source of the virus for the recurrent annual mass mortality events that are a feature of POMS [41]. A sudden increase in water temperature was used to try to trigger viral reactivation because a seasonal increase in water temperature is a major risk factor for POMS and temperature thresholds have been demonstrated for infection *per se* and for disease [17,35,38,44].

We did not observe OsHV-1 mortality (objective 1) or viral transmission to naïve spat (objective 2) in this study when adults carrying the virus were thermally manipulated. Around 2% of the population had detectable OsHV-1 DNA in the gill and mantle one month before these oysters were recruited into the experiment. Similarly, de Kantzow et al. [48] did not observe a second episode of mortality by increasing the water temperature from 18 °C to 22 °C in a group of oysters that had survived an OsHV-1 injection 14 days earlier and that still had a high prevalence of OsHV-1 infection. The results of both studies mirror those of an experiment that was conducted in France in which there was no mortality when oysters that had been exposed to OsHV-1 during a natural outbreak at 18 °C were held at 13 °C for 40 days and then placed at 20 °C [35]. However, in a subsequent experiment in France, oysters that had been exposed to OsHV-1 and then been kept in cool water in a similar way to the previous experiment did experience high mortality after the water temperature was increased to 21 °C [39]. Recently, reactivation of apparent latent OsHV-1 infection was demonstrated in *C. gigas* [49]; the oysters were injected intramuscularly with a high dose of OsHV-1, kept at 20–22 °C and the survivors were maintained for 21 days, by which time OsHV-1 could not be detected in their tissues. Then, they were injected with sodium butyrate and placed in seawater containing the immune pathway inhibitor thiophenecarboxamide at 30 °C for 6 h; this was soon followed by viral replication in oyster tissues and shedding of OsHV-1 into seawater [49]. The extreme stress imposed in that experiment makes it hard to compare with the others. Across these experiments, there were differences in experimental models including the age and size of oysters, exposure conditions, environmental parameters and timelines for exposure–temperature change–reexposure, all of which may influence the outcomes.

The third objective of this study was to determine whether previously exposed adult oysters were susceptible to reinfection with OsHV-1 leading to mortality, that is, whether they had some form of immunity to the virus. That was the case because very high cumulative mortality occurred in naive oysters compared to low mortality in those that had already been exposed to OsHV-1 and survived. When tested in August 2016, one month before recruitment into this experiment, the prevalence of detectable OsHV-1 infection in the PEAd oysters was 2% and viral loads were below the level of quantification of the qPCR assay (see materials and methods). These observations suggest that the prior exposure to OsHV-1 was protective. But, could other factors explain these results? Specifically, were there any differences in the known, major risk factors between the two treatment groups, such as differences in water temperature, genetics, age or size? Water temperature is an important risk factor for OsHV-1 disease expression [35,38] but can be dismissed here because the temperatures in each treatment were similar (Table 2). Concerning genetics, although *C. gigas* can be selected and bred for resistance to OsHV-1 [50], the non-exposed and previously exposed oysters used in this study were from hatchery batches (SPL14B, SPL13B) that were not expected to differ in genetic resistance to OsHV-1 because selected oysters were not available in Australia at that time. Following reports on the effects of both age and size from Europe [11] and Australia [12,13], Hick, Evans, Rubio, Dhand and Whittington [15] conducted detailed experiments using 17-month-old, approximately 60 mm (shell length) (small) and 69 mm (large) oysters from the hatchery batch SPL13B to evaluate the effects of age and size on susceptibility to OsHV-1. Under natural exposure, size did not affect susceptibility; however, in the laboratory infection model, the hazard of death was 2.3 times higher for the small adult oysters [15]. The previously exposed adult oysters (PEad) in experiment 2 in the present study were older (27–37 months, 123 mm), but assuming similar age and size-based susceptibility to those in the study of Hick, Evans, Rubio, Dhand and Whittington [15], they would be expected to have approximately 2 times lower susceptibility to OsHV-1 than the non-exposed adults (NEad), which were smaller (36 months, 75 mm). In fact, the observed hazard of death was 119 times lower for the previously exposed group, which is most unlikely to be due to the difference in size. This is a substantial effect, especially considering the larger dose of OsHV-1 administered to the previously exposed group, because there is a strong, direct, dose–response effect in this laboratory infection model [20].

The findings in this study confirm those of de Kantzow, Whittington and Hick [48] who used the same infection model to show that oysters challenged with OsHV-1 at a water temperature of 22 °C had about one fifth the risk of death if pre-exposed to OsHV-1 at 18 °C, a temperature at which OsHV-1 infection occurs without mass mortality. Considering the prior results [24,48] together with those of the present study, non-lethal exposure of *C. gigas* to OsHV-1 in a natural estuary setting or in a laboratory infection model can reduce the mortality of the same oysters subjected to subsequent exposure to the virus, in either a natural setting or in the laboratory. There would appear to be a consistent pattern in different environments, manifested as resistance to the effects of an otherwise lethal OsHV-1 exposure in oysters that previously have been exposed to the virus and survived.

The underlying mechanisms for the profound reduction in susceptibility of the previously exposed oysters were not investigated in this study and the experimental design precludes an assessment of whether the observed immunity was intrinsic or it was induced in individual oysters by their first exposure to the virus. This is because the oysters were the survivors of prior mortality events, and therefore had been recruited into the experiment after displaying a resistant characteristic. However, exposure to the virus *per se* may be more important than surviving a mortality event. This was shown by de Kantzow, Whittington and Hick [48] who demonstrated resistance after pre-exposure to OsHV-1 without mortality.

Oysters have innate immune response capacities such as phagocytosis, autophagy, apoptosis, humoral antimicrobial/antiviral peptides, the JAK/STAT pathway, RNA interference and receptors for pathogen-associated molecular patterns (PAMPs), many of which are relevant in herpesvirus infection in *C. gigas* [51,52,53,54,55,56,57]. Breeding from the survivors of natural OsHV-1 exposure events (mass selection) was reported to be a successful disease mitigation strategy in France, indicating that immune individuals have heritable traits [58]. Experimentally, inoculation of *C. gigas* with polyinosinic:polycytidylic acid (poly I:C), which is a synthetic double-stranded RNA used to stimulate anti-viral immune responses, evokes an interferon response which protects against subsequent challenge with OsHV-1 and the protection could be transmitted vertically to progeny [59,60]. Poly I:C, inactivated OsHV-1 and OsHV-1 proteins stimulated reactive oxygen species production and upregulation of immune-related genes ex vivo in *C. gigas* hemocytes [61]. Both intra- and inter-generational immunity to OsHV-1 could be explained by these pathways of innate immune stimulation.

OsHV-1 infection has been found in survivors after disease outbreaks at other locations in the world, for example in France [62], Mexico [63] and California [64]. This may be a consistent phenomenon leading to the development of natural resistance at the population level. OsHV-1 has also been detected in *C. gigas* in the absence of recurrent, seasonal mortalities, mainly in Asia [27,28,65]. This is the geographic origin of *C. gigas*, which was translocated to Europe, Oceania and the Americas for aquaculture, and is hypothesised to be the origin of OsHV-1 [66]. In Asia, the longer period of association between OsHV-1 and *C. gigas* may have led to the harmonious co-existence of the pathogen and host through the evolution of host immunity.

## 5. Conclusions

Adult *C. gigas* can carry OsHV-1 DNA (a proxy for infection) for lengthy periods and while the reactivation of viral replication leading to mortality and transmission of the virus to naïve oysters may be possible, it may require specific conditions that were not present in the current experimental design. However, these oysters were not very susceptible to experimental reinfection with OsHV-1 and consequently appeared to possess immunity to the virus. Non-lethal exposure of *C. gigas* to OsHV-1 either naturally or in the laboratory is followed by apparent immunity to the effects of the virus on subsequent exposure, in both the estuary and the laboratory. There would appear to be a consistent pattern of stimulation of robust immunity through non-lethal exposure to OsHV-1 in different environments. Further investigation is required to evaluate the mechanisms responsible for resistance to disease in oysters previously exposed to OsHV-1, whether they can be exploited commercially to prevent POMS outbreaks or to determine the source of the virus for recurrent seasonal outbreaks.

## Figures and Tables

**Table 1:** Results of experiment 1 showing the number of oysters that tested positive for OsHV-1 DNA on day 12 and the water temperature (mean ± standard deviation).

System	Type of Oyster	Number of Oysters Sampled on Day 12	Number Positive for OsHV-1 DNA ^a^	Viral Load ^b^	Water Temperature Days 1 to 6	Water Temperature Days 7 to 12
1	PEad	12	0	-	16.0 °C ± 0.3 °C	23.1 °C ± 0.9 °C
	NEsp1	100	0	-		
2	PEad	12	2	bloq	15.3 °C ± 0.3 °C	22.4 °C ± 0.9 °C
	NEsp1	100	0	-		
3	PEad	12	2	bloq	15.4 °C ± 0.3 °C	22.6 °C ± 0.9 °C
	NEsp1	100	0	-		
4	PEad	36	1	bloq	15.6 °C ± 0.2 °C	15.7 °C ± 0.3 °C
	NEsp1	100	0	-		

^a^ spat were processed in pools. ^b^ bloq: below the limit of quantification of the qPCR assay.

**Table 2:** Water temperature in experiment 2 (mean ± standard deviation).

System/Tank	Oysters	Challenged with OsHV-1	Water Temperature Days 1 to 10
1	PEad	Yes	23.4 °C ± 0.4 °C
2	PEad	Yes	22.9 °C ± 0.4 °C
3	PEad	Yes	22.7 °C ± 0.3 °C
4	NEad	Yes	23.0 °C ± 0.4 °C
SAT 1	NEsp2	Yes	22.1 °C ± 1.4 °C
SAT 2	NEad	No	22.0 °C ± 1.0 °C

SAT, standalone tank.

**Table 3:** Results of experiment 2 showing the oysters sampled, the number positive for OsHV-1 DNA and viral load.

Treatment (Oysters Used)	Day Post Injection	Live or Dead	Aquarium System	No. Sampled	No. Positive OsHV-1 PCR	Viral Load ^a^		
						Geometric Mean	Lower 95% CI	Upper 95% CI
Previously exposed adult oysters (PEad) challenged with OsHV-1	2	dead	2	1	1	2.14 × 10^4^	-	-
	7	dead	3	1	1	1.74 × 10^4^	-	-
	9	dead	3	1	1	4.41 × 10^2^	-	-
	10	dead	1	1	1	1.56 × 10^3^	-	-
	4	live	1	12	10	4.44 × 10^2^	3.30 × 10^2^	5.96 × 10^2^
	4	live	2	12	9	1.67 × 10^3^	1.08 × 10^3^	2.67 × 10^3^
	4	live	3	12	11	8.91 × 10^2^	7.01 × 10^2^	1.13 × 10^3^
	10	live	1	11	9	6.69 × 10^1^	4.46 × 10^1^	9.40 × 10^1^
	10	live	2	11	9	9.58 × 10^1^	8.76 × 10^1^	1.05 × 10^2^
	10	live	3	10	8	6.55 × 10^1^	5.21 × 10^1^	8.23 × 10^1^
Non-exposed adult oysters (NEad) challenged with OsHV-1	2	dead	4	13	13	2.23 × 10^5^	6.03 × 10^4^	2.54 × 10^5^
	3	dead	4	6	6	1.69 × 10^5^	3.15 × 10^3^	2.52 × 10^5^
	4	dead	4	2	2	8.84 × 10^4^	4.60 × 10^3^	1.70 × 10^6^
	4	live	4	3	2	2.50 × 10^3^	7.14 × 10^2^	8.77 × 10^3^
Positive control (NEsp2) challenged with OsHV-1	2	dead	SAT 1	2	2	3.26 × 10^5^	2.05 × 10^5^	5.18 × 10^5^
	3	dead	SAT 1	7	7	1.31 × 10^5^	5.12 × 10^3^	1.82 × 10^5^
	4	dead	SAT 1	1	1	3.37 × 10^5^	-	-
	4	live	SAT 1	2	2	8.39 × 10^2^	1.16 × 10^2^	6.07 × 10^3^
Negative control (NEad)	4	live	SAT 2	6	0	-	-	-
not exposed to OsHV-1	10	live	SAT 2	6	0	-	-	-

^a^ OsHV-1 DNA copies/mg tissue; CI, confidence interval; SAT, standalone tank.

## Data Availability

Original data are available upon reasonable request to the corresponding author.
